# A Genetic Attack Against Machine Learning Classifiers to Steal Biometric Actigraphy Profiles from Health Related Sensor Data

**DOI:** 10.1007/s10916-020-01646-y

**Published:** 2020-09-15

**Authors:** Enrique Garcia-Ceja, Brice Morin, Anton Aguilar-Rivera, Michael Alexander Riegler

**Affiliations:** 1 SINTEF Digital, Oslo, Norway; 2 Barcelona Supercomputing Center, Barcelona, Spain; 3 SimulaMet, Oslo, Norway

**Keywords:** Impersonator attack, Biometric profiles, Machine learning, Genetic algorithms

## Abstract

In this work, we propose the use of a genetic-algorithm-based attack against machine learning classifiers with the aim of ‘stealing’ users’ biometric actigraphy profiles from health related sensor data. The target classification model uses daily actigraphy patterns for user identification. The biometric profiles are modeled as what we call *impersonator examples* which are generated based solely on the predictions’ confidence score by repeatedly querying the target classifier. We conducted experiments in a black-box setting on a public dataset that contains actigraphy profiles from 55 individuals. The data consists of daily motion patterns recorded with an actigraphy device. These patterns can be used as biometric profiles to identify each individual. Our attack was able to generate examples capable of impersonating a target user with a success rate of 94.5*%*. Furthermore, we found that the *impersonator examples* have high transferability to other classifiers trained with the same training set. We also show that the generated biometric profiles have a close resemblance to the ground truth profiles which can lead to sensitive data exposure, like revealing the time of the day an individual wakes-up and goes to bed.

## Introduction

The use of wearable devices, sensing technologies and machine learning methods to monitor and predict user behavior has gained a lot of attention in recent years. It has been shown that these technologies have great potential to solve many relevant problems such as continuous mental health monitoring [22], elderly care assistance [44], cancer detection [26] and sports monitoring [4]. Many of those works use wearable sensors data, such as accelerometers, gyroscopes, temperature or heart rate, to train machine learning models. Being already embedded in many types of devices like smartphones, smart-watches and fitness bracelets, accelerometers are the most common wearable sensors. Actigraphy devices record human motion levels using accelerometers. Usually, these devices are worn on the wrist like a regular watch and are very popular in medical studies due to their non-invasive nature. Actigraphy devices can be used to monitor sleep patterns [25] or predict depression states [21], among other things.

Recent works have suggested that such devices can be used to capture user behavior to build *biometric profiles*, i.e., behaviour signatures that uniquely identify someone, similar to a fingerprint. These types of systems can be used for continuous user authentication [1]. Some of those user authentication systems are based on hand gestures [10, 57], finger-snapping [11], touchscreen interactions [27] and gait analysis [28]. Several research works have also explored the idea to use behaviour analysis with smartphones for user authentication [32, 34, 39, 50].

Most of the aforementioned biometric systems rely on machine learning algorithms to perform the user authentication [36]. Recently, it has been shown that such algorithms are prone to different types of attacks [8, 31]. This fact is of paramount importance to authentication systems since bypassing them, means that the attacker could have access to sensitive personal data.

Most of the research works in machine learning applied to the medical field [15, 23, 45], focus on developing accurate models in terms of prediction capabilities, however, security aspects are usually left aside. Some points why security aspects also need to be considered in medical domain applications are (i) medical data is shared more frequently and we have to make sure to protect it properly, (ii) even fully anonymized data might be giving hints about patients or demographics, (iii) model sharing is quite common in the field of AI and seen as save but we show that one might be able to extract sensitive information, and (iv) from reverse engineering these models we can also learn more about how they work and thus, increase explainability and trust which is important for AI in medicine.

In this work, we propose the use of a genetic algorithm based attack against machine learning classifiers with the objective of ‘stealing’ users’ biometric profiles. The biometric profiles are modeled as *impersonator examples* which are generated by repeatedly querying the target classifier. These examples can then be used to impersonate a given user. Furthermore, we show that the *impersonator examples* are transferable to other models. This means that the same example can be used on a different type of classifier, provided that the new classifier was trained with the same training set as the original one. Our proposed approach assumes a *black-box* setting in which the type of classification model is unknown to the attacker. While previous works mainly focus on computer vision systems, to the best of our knowledge, this is the first research work that develops an attack against a biometric actigraphy system. In summary, the main contributions of this paper are:

- We propose the use of a genetic algorithm based approach to attack a classifier with the objective of generating actigraphy biometric profiles.
- We introduce the new concept of *impersonator examples*, generated biometric profiles that are able to impersonate a given user. Our impersonator examples achieved a high success rate on actigraphy data.
- We demonstrate the effectiveness of the attack at different strength levels in a *black-box* scenario where the attacker does not know the type of classifier.
- We show that the generated *impersonator examples* are highly transferable to other types of classifiers, exposing potential vulnerabilities in the system.
- We show that the generated *impersonator examples* can resemble the real biometric profiles, thus, having the potential to expose sensitive user information.
- We empirically demonstrate that by omitting confidence scores on the predictions, the attack can be mitigated to a great extent.

The remainder of this paper is organized as follows: Section “Related work” explains the related work about different types of attacks against machine learning classifiers. We also provide a brief background about genetic algorithms. Section “Dataset and feature extraction” describes the dataset used in the experiments. Section “User recognition classifier: The target model” describes the classification model subjected to the attack, while the threat model and the method to generate impersonator examples are introduced in Section “Threat model”. The results are presented and discussed in Section “Experiments and results”. Conclusions appear in Section “Conclusion”.

## Related work

Machine learning has permeated our society by providing services and support in almost every field. In many cases, it provides critical services, thus, making them attractive to potential attackers (*adversaries*) wanting to disrupt the system or take advantage from it. It has been shown that machine learning algorithms are vulnerable to different types of attacks and under different settings. In the following, we provide an overview of different types of attacks to machine learning classifiers and related work in context to them.

### Model extraction attacks

For these types of attacks, the adversary aims to duplicate the functionality of the target model. For example, Tramèr et al. [55] showed how different types of classifiers such as decision trees, logistic regression, SVM and deep neural networks can be ‘copied’ with high fidelity by querying them and using the returned predictions’ confidence scores.

### Membership inference attacks

The objective of this type of attack is to determine if a given data point was part of the target model’s training set [51]. Pyrgelis et al. demonstrated how these types of methods can be used to determine if a user was part of the training set of location time-series records which leads to privacy-related vulnerabilities [42].

### Adversarial examples attacks

Goodfellow et al. [24] define an adversarial example as ‘*...inputs formed by applying small but intentionally worst-case perturbations to examples from the dataset, such that the perturbed input results in the model outputting an incorrect answer with high confidence.*’. In computer vision tasks, it is often the case that the methods try to reduce the perturbations. For example, these small perturbations can make two images being perceived as the same by the human eye but a classifier can output very different results. Recently, this has been a very active research area with several proposed attack and countermeasure methods, specially in the computer vision field [37, 54]. An extreme case is the one presented by Su et al. in which they were able to craft image adversarial examples to mislead a classifier by changing a single pixel [53]. Alzantot et al. [3] showed that genetic algorithms can be used as an alternative to gradient-based methods to generate adversarial examples on image datasets.

### Data extraction attacks

Here, the objective is to reconstruct training data points by just having access to the trained model. Fredrikson et al. developed an inversion attack and showed how to recover face images given only the users’ name and access to the model’s predictions [16]. Song et al. demonstrated that it is possible to modify training algorithms such that information from the training set can be leaked afterwards [52]. They also performed their tests on a face images dataset.

### Impersonate attacks

In this type of attack, the adversary generates a biometric profile that allows her/him to be identified/authenticated as some other predefined person. Sharif et al. proposed an attack that works on a *white-box* setting that consists of printing a pair of glasses that allows an adversary to impersonate another individual [48]. Quiring et al. proposed a method to modify programmers’ source code to mislead authorship attribution [43]. That is, they transformed the code from author *A* to look as if it was written by author *B* and achieved a success rate of 81.3%.

For a more complete list of types of attacks, the reader is referred to two excellent surveys [8, 31]. In this work we focus on impersonate attacks. Most of the previous works about impersonation target computer vision systems, in particular, face recognition [7, 48, 49]. Contrary to previous works, we evaluate our method on actigraphy biometric profiles. While some of the previous works conducted their tests in a *white-box* setting, here, we consider a *black-box* scenario where the model is unknown but can be queried and prediction confidence scores are available.

### Genetic algorithms

Genetic algorithms (GAs) are a nature-inspired computing technique based on Darwinian evolution. Possible solutions to a problem are (usually) encoded into binary strings that represent the DNA of a particular individual interacting with its environment. A population of individuals is randomly initialized and then is subject to a selection process, where the fittest ones have an increasing probability of reproducing. Selected individuals are combined using genetic operators (i.e., selection, crossover and mutation), and the process is repeated for several generations until the stop criteria are met. Fitness is computed using an objective function, which is particular to the problem and reflects solution quality. GAs are a global optimization approach based on implicit parallelism [5].

During each generation, selection, crossover, and mutation are applied repeatedly to the individuals in the population. Selection determines which individuals will be used to produce the next generation. This is usually a probabilistic process where the best individuals have higher probability to pass their schemes to the offspring. Tournament, and roulette wheel are some examples of selection methods commonly used in GAs [12, 14, 33].

Crossover is the main devise used to recombine the selected solutions. In crossover, large sections of the chromosome are interchanged between the parents. Crossover can occur in one or many points of the chromosome [30]. On the other hand, mutation is a punctual, random modification to the chromosome. This operation is used to enforce diversity and avoid local optimum. Several methods are proposed in the literature to perform these operations. Besides, abstraction of genetic operators into sampling of probability distributions is also possible to solve hard problems [40].

GAs have been used to solve a large variety of problems. Their application to biometric systems is reported in the literature. For example, GAs have been used on iris image reconstruction from binary templates [17]. Galvan et al. [18] used GAs to perform feature selection for a depression detection model based on actigraphy signals.

In regards of biometric systems attacks (a.k.a. spoofing), GAs have been reported to be used for impersonation in speaker recognition systems [2] and iris recognition systems [29]. In the first case, GAs were used to generate artificial signals of short duration but with high recognition score. These signals could not account as human speech, but they were effective spoofing devices. The latter reference describes impersonation methods like fake contact lenses and flat prints of the original irises. The work of Galbally et al. [17] and the potential of GAs to generate synthetic iris images are also mentioned in this reference.

Other examples are the work of Pereia et al. [41], who proposed GAs for feature extraction in a fingerprint spoof detection system. Moreover, Nguyen et al. [35] used GAs to create artificial images that are classified with high confidence as familiar objects by deep neural networks. Although, they were unrecognizable to the human eye.

Based on this review, the application of GAs for impersonation of actigraphy profiles seems a subject open to further research.

## Dataset and feature extraction

To validate our approach, we used the *DEPRESJON dataset* which is publicly available [20]. The dataset was collected by 55 participants wearing an actigraphy watch^1^ on their right wrist. On average, the participants wore the device for 20 consecutive days. The device captures activity levels using a piezoelectric accelerometer that captures the amount and duration of movement in all directions. The sampling frequency is 32Hz, and movements over 0.05 g are recorded. A corresponding voltage is produced and is stored as an activity count in the memory of the device. The number of counts is proportional to the intensity of the movement. The accumulated activity counts are continuously recorded in one minute intervals. The dataset contains information about the mental condition of each participant (depressed/non-depressed). For our experiments, we did not use mental condition information since the aim of the classifier is to perform user identification based on activity level patterns as in our previous work [19].

From the raw activity counts, 24 features were extracted in a per day basis. The features are the mean activity counts for each hour of the day (*h*1 − *h*24). Missing values were set to − 1 and the first day from each user was excluded since it usually began at midday, thus, having a lot of missing values before that. The features were normalized between 0 and 1. After feature extraction, the total number of instances (feature vectors) were 1089, each, representing a single day of data.

## User recognition classifier: The target model

In this section we describe the *target model*, i.e., the classifier from which we will acquire the user actigraphy profiles. The classification model is trained on user actigraphy daily profiles encoded as 24 features. Each feature represents the mean activity level for each hour of the day. As classification model, we used a Random Forest [9] with 500 trees since it has been shown to produce good results for different applications [13]. The target class we chose to predict for this model is the respective user id. To test the performance of the user recognition classifier we ran 10 iterations. In each iteration, we trained the model with 50*%* of each users’ data and used the remaining 50*%* as test set. For more details the reader is referred to our previous work [19]. Table 1 shows the recognition results.

**Table 1:** Classifier performance results

Metric	Value
Accuracy	0.63
True Positive Rate (TPR)	0.63
True Negative Rate (TNR)	0.99
Balanced accuracy	0.81

**Fig. 1:**
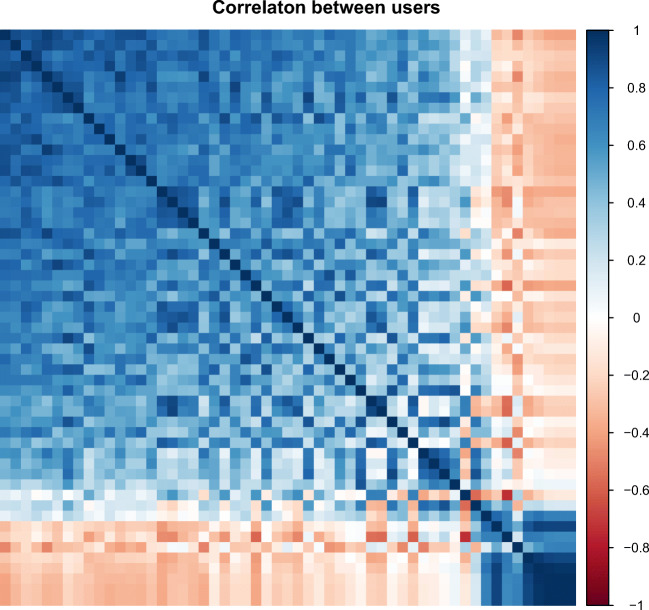
Correlation between users’ activity levels

From these results, it can be seen that the true positive rate is 0.63. Even though these results are not perfect, they demonstrate the difficulty of the problem to identify users solely based on their daily movement patterns. Part of this challenge is attributed to the fact that many humans have very similar daily patterns. Many of them start activities early in the morning and go to sleep during night. This fact is also depicted in Fig. 1, which shows the correlation between each pair of users from their hourly mean activity levels. From this plot, it can be observed that most users have a high positive correlation with the others, making user identification into a challenging problem. Since in this work the focus does not lie on the task of training highly accurate user recognition models but on a method to ‘steal’ user biometric profiles given an already trained model we are not concerned about the accuracy of the model.

In the following, we will formally define the problem of generating *impersonator examples* by repeatedly querying the previously described model. The obtained *impersonator examples* can then be used to impersonate a given user.

## Threat model

A threat model is a definition of the characteristics of the system under consideration. This includes the target model’s specification and also encompasses the information the adversary has and the actions she/he is capable of [38]. Here, we consider a setting with a *target model* *F*, a *target user* *t* and an *attacker* *A*. The target model *F* is a function:

1 $$  f(x,t) \rightarrow [0,1] $$

where *x* is a biometric profile coded as a real-valued feature vector. The target model outputs a score between 0 and 1 that represents the confidence that the biometric profile *x* belongs to the target user *t*. The objective of the attacker is to impersonate the target user. We consider a *black-box* setting in which the attacker does not know what type of classifier *F* is. The attacker influence is exploratory [8] since she/he can only manipulate the test data (queries). We assume that the attacker knows the input structure and domain. In this case, the input is 24-dimensional feature vectors with values between 0 and 1. The aim of the attacker is to find a biometric profile *x*^∗^ for the target user *t* such that *f*(*x*,*t*) is maximized. Formally, this can be defined as:

2 $$  x^{*} = \operatorname*{argmax}_{x \in [0,1]^{d}} f(x,t) $$

Where *x*^∗^ is the *impersonator example* and [0,1] is the set of real-valued vectors between 0 and 1 with dimension *d*. Thus, an *impersonator example* can be defined as *“a biometric profile that was not produced by the target user but was synthetically generated with the aim of impersonating that target user”*. The main difference with an adversarial example is that the latter belongs to an initial predefined class *c* and the objective is to make the model classify it as another class *c*^′^,*c*^′^≠*c*. In the case of an *impersonator example*, there is no initial class assigned to the data point.

### Generating impersonator examples

**Fig. 2:**
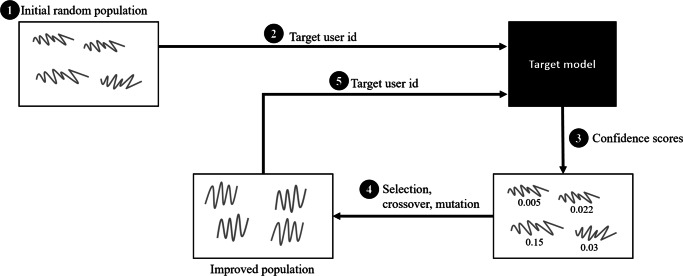
Steps to generate impersonator examples. **1.** Start with an initial random population, 4 in this case. **2.** Query the target model using the initial random population. **3.** Obtain the confidence scores from the target model for each individual. **4.** Perform selection, crossover and mutation operations on the individuals based on the confidence scores to generate an improved population. **5.** Query the target model with the improved population and repeat steps 3-5 until there is no improvement or the max number of allowed iterations has been reached. The final impersonator example is the individual with the highest fitness value

In this section we describe the procedure to generate *impersonator examples* using genetic algorithms, i.e., find *x*^∗^ (2). Figure 2 depicts the steps needed to generate an *impersonator example* by issuing queries to the target model *F*.

1. Generate an initial random population of *n* individuals (solutions). In this case, each individual is a feature vector of size 24 with each value representing the mean activity level for that hour of the day. The initial values are drawn from a uniform distribution between 0 and 1.
2. For each individual *i*, query *F*(*i*,*userid*) using the *target user id*.
3. Obtain the resulting confidence scores. This is the fitness function.
4. Perform the corresponding genetic operations: selection, crossover and mutation to generate an improved population.
5. Use the improved population to query *F* again. Repeat steps 3 to 5 until the max number of iterations has been reached or if there is no improvement after 100 iterations.

The resulting *impersonator example* is the individual with the highest fitness function from the last iteration.

## Experiments and results

**Fig. 3:**
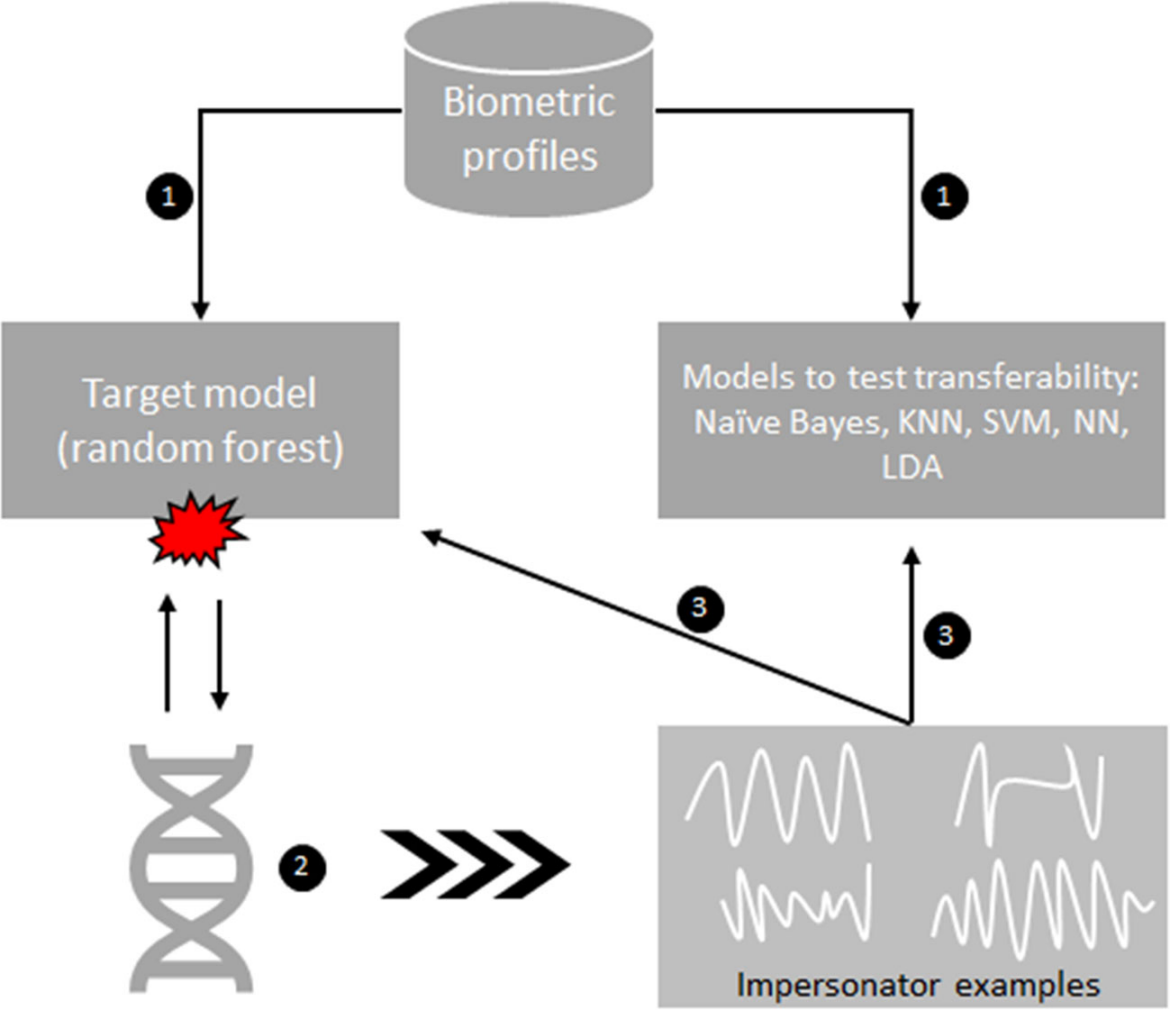
Experiment workflow. **1.** The biometric profiles are used to train the target model and additional models that will be used to test transferability. **2.** The proposed method is used to attack the target model and generate impersonator examples. **3.** The impersonator examples are used to impersonate the users on both, the target model and the models used to test transferability

In this section, we describe the conducted experiments to validate the proposed approach. We also test the transferability of the generated *impersonator examples* to other classifiers. Finally, we performed experiments on a target model that does not output confidence scores. Figure 3 shows a high level schematic view of the process.

To evaluate the effectiveness of the attack, we tested three different settings by increasing the ‘strength’ of the attack. Namely, we tried *weak, medium* and *strong* attacks. The attack’s strength is parametrized by the genetic algorithm’s population size. The *weak, medium* and *strong* attacks have population sizes of 10, 20 and 50, respectively. Increasing the population size generates more diverse individuals, thus, increasing the chances of avoiding local maxima. The maximum number of generations was set to 1500. The second stop criterion is if there is no increase in the fitness function for the last 100 generations the process stops. We used the genetic algorithms R library *GA* [46, 47] with the default values for the following parameters. The probability of crossover was set to 0.8. The probability of mutation is 0.1. *Elitism*, the number of best fitness individuals to survive at each generation is set to the top 5*%*. The experiments were run on a personal computer with 32 GB of RAM and an Intel i7-8850H CPU with 12 logical cores. The GA procedure was run with the *parallel* parameter set to 10.

Table 2 shows the averaged results for all target users for the three types of attacks and their corresponding success rates (SR). The SR is the percentage of users that were correctly classified by using the *impersonator example*. Since the probability of guessing the right answer by random is 0.018, i.e., 1 out of 55 users in the database, we set a *confidenceThreshold* = 0.20 which is a lot higher than a random guess. Thus, in order to increase the confidence of the classification, an *impersonator example* is considered to be successful only if it is classified as the target user it tries to impersonate and if the confidence score produced by the target model is ≥ *confidenceThreshold*.

**Table 2:** Results for the three genetic attacks with incremental strength

	population size	avg. generations	avg. sec.	SR
Weak attack	10	841	30	76.3%
Medium attack	20	922	53	91.0%
Strong attack	50	1019	116	94.5%

From these results, we can see that as the strength of the attack increases, so does the success rate (SR). However, the average number of generations and the average time in seconds also increases. This translates into the number of queries to the target model which is the *population size* * *number of generations*. Even with the weak attack, the SR is relatively high, i.e., 42 out of the 55 users were successfully impersonated. For the strong attack, 52 out of the 55 users were successfully impersonated and on average, it took approximately 2 minutes to perform the attack for a given target user. One thing to note is that the SR of the attack was higher than using the real test set examples from the users (see Table 1).

**Fig. 4:**
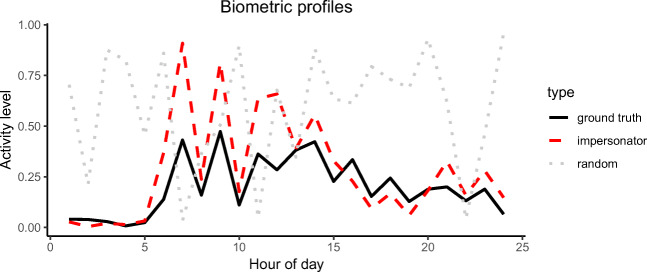
Biometric profile of the initial random solution, ground truth and resulting impersonator example for the target user *control10*

Figure 4 shows the biometric profiles for one of the target users (control10). The point line shows the initial solution generated at random. The solid line depicts the ‘ground truth’ profile, which was obtained by averaging the user’s training data. The generated *impersonator example* is represented by the dashed line. It can be observed that the *impersonator example* follows an approximate trend compared to the ground truth, however at some points the former has much higher peaks. Not having an exact or close biometric copy of the ground truth was expected since the latter is a representation of the average of all the target user’s data points. Furthermore, the ground truth is independent of other users whereas the *impersonator example* is generated by taking into account all other users indirectly through the fitness function. Still, the *impersonator example* looks very similar to the average biometric profile of the target user.

**Fig. 5:**
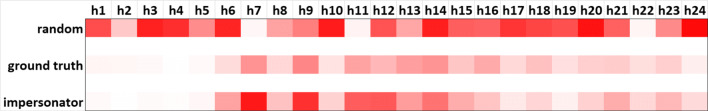
Heatmap of the initial random solution, ground truth and resulting impersonator example for the target user *control10*. h1...h24 are the hours of the day

Figure 5 shows the same information but as a heat map with higher values being more opaque. With this type of representation, it is even possible to guess at what time of the day the target user woke up and and what time she/he went to sleep. Here, it seems that the user woke up around 6 a.m and went to sleep around midnight, however we cannot confirm this since the dataset does not provide this information explicitly. This shows the potential of the *impersonator examples* not just to impersonate someone but also to expose sensitive information about the target user.

### Transferability

In the context of adversarial examples, *transferability* is defined as the capacity of an adversarial example crafted for one model, to be used to attack another model as first shown by Biggio et al. [6]. To this extent, we now evaluate the transferability of the *impersonator examples* generated from the previous target model and use them on new models trained with the same training data set. For this evaluation, we only used the successful *impersonator examples* generated by the *strong attack* from the previous experiment (52 in total).

To test transferability, we trained five different classifiers: Naive Bayes, K-nearest neighbors (KNN), Support Vector Machine (SVM), Neural Network, and a Linear Discriminant Analysis (LDA) classifier. Since each classifier generates confidence scores differently, we tested the success rate *with* and *without* a *confidenceThreshold* = 0.20. For the case with confidence threshold, an *impersonator example* is considered to be successfully transferable if the predicted user is the same as the one it tries to impersonate and the returned confidence score from the model is ≥ *confidenceThreshold*. Table 3 shows the transferability success rates. Here we can see that the *impersonator examples* were least successful against the SVM with a success rate of just 2.0*%* and 33*%* *with* and *without* confidence threshold. The most successful attack was against the KNN classifier when *k* = 1 with a success rate of 96%, i.e., 50 out of 52 examples succeeded in their attack. As the number of neighbors *k* was increased, the success rate started to decrease. Another thing to note is that KNN was the most vulnerable classifier but at the same time, it means that it was the best at classifying new data points. We hypothesize that this is because it has been shown by Xi et al. that a KNN with *k* = 1 and with Dynamic Time Warping as a distance metric, is “exceptionally difficult to beat” for time series classification problems [56]. Here, an impersonator example is a 24 size feature vector where each entry represents the hour of the day, thus, it is a time series. Dynamic Time Warping is a method used to ‘align’ shifted time series. In this case, the impersonator examples are already aligned in time because the feature vector at position 1 corresponds to the first hour of the day, the second position to the second hour of the day and so on. The classification of impersonator examples problem is equivalent of classifying time series data where the time series are already aligned, thus, based on the work of Xi et al. [56], a one-nearest-neighbor model is expected to excel in this task.

**Table 3:** Transferability success rates

	With confidence threshold	No threshold
Naive Bayes	61.5%	61.5%
KNN, k = 1,3,5	96.0%,73.0%,69.0%	96.0%,73.0%,69.0%
SVM	2.0%	33.0%
Neural Network	11.5%	23.0%
LDA	57.7%	64.0%

The reason for the big differences of some classifiers when using or not a threshold is because some of them return more uniformly distributed confidence scores than others such as the SVM. On the other hand, some classifiers return more ‘crisp’ scores. For example, the confidence score returned by KNN when k is set to 1, is either 1 or 0. This is because it computes the score as the percentage of neighbors that agreed with the final class label. All in all, we can see that the generated *impersonator examples* have very high transferability with some classifiers.

### Omitting confidence scores

An easy to implement countermeasure to this type of attack would be to omit the confidence scores that the target model outputs. To test the effectiveness of this defensive measure, we performed a *strong attack* against the same target model, but this time it only returns a binary decision as the classification result instead of including confidence score information. Table 4 shows the obtained results. In this case, the success rate was 1.8%, i.e., the attack was able to impersonate just 1 of the 55 users which is equivalent of guessing at random. Also, it was more computationally intensive. This counter measure was able to limit the attack’s capability almost entirely. The study of countermeasures when confidence scores are still present as part of the output will be left as future work.

**Table 4:** Results for the strong genetic attack when the target model omits confidence scores

	Population size	avg. generations	avg. sec.	SR
Strong attack	50	1500	302	1.8%

### Failure analysis

**Fig. 6:**
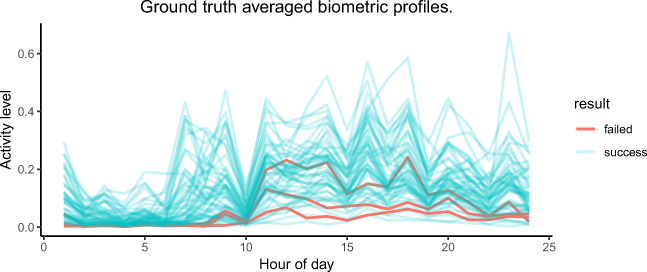
Ground truth biometric profiles of failed and successful impersonated users. (For a colored version of this plot please see the online version)

After analyzing the results, we noted that the method failed on three users: condition1, condition20 and condition21 in all of the three attacks (weak, medium, strong). We hypothesized that this might be due to irregular daily patterns. However, by visually comparing the patterns between successful v.s. failed impersonated users, we could not find any ‘out of the normal’ deviations (see Fig. 6). For two of the failed cases, their activity levels seem low, but still are within the normal limits compared to others. Our second hypothesis was that the GA search was stuck in a local maximum. Thus, we increased the population size from 50 to 100 and increased the max iterations from 1500 to 2000. This time, the three users were successfully impersonated. This rises the following research question: *What data/model properties make some users more vulnerable than others?* This question would require different experiments to be answered, and is out of scope of the present work. Nevertheless, the research question itself is an interesting finding and will be tackled in future work.

### Source code and data

The data and R code used in our experiments have been made publicly available. The code contains the necessary files to run the experiments and produce the figures. The processed dataset and code can be downloaded from: https://github.com/enriquegit/impersonator_attack_ga. The original dataset used in this work was introduced in [20]. The experiments were conducted on a PC running Windows 10 and R version 3.5.1.

## Conclusion

In this paper, we proposed the use of a genetic algorithm based approach to attack a classifier with the objective of generating actigraphy biometric profiles. We introduced the concept of *impersonator examples* which are generated biometric profiles that are able to impersonate a given user. We could show that our impersonator examples are able to achieve a high success rate (94.5*%*) on a publicly available actigraphy dataset. Furthermore, we demonstrated the effectiveness of the attack at different strength levels in a *black-box* scenario where the attacker does not have information about the type of the target model. In addition, we demonstrated that the generated *impersonator examples* are highly transferable to some other types of classifiers, opening the door for more system vulnerabilities. We also showed that the generated *impersonator examples* can resemble the real biometric profiles, thus, having the potential to expose sensitive user information. We also showed that a countermeasure such as omitting confidence scores is very efficient in reducing the success rate of the attack from 94.5*%* to 1.8*%*.

For future work, apart from the additional research question that we defined during the failure analysis, we plan to explore the generalisability of the approach on other sensor based datasets. For example, datasets that utilised Apple smartwatches. Furthermore, we plan to explore how robust multimodal datasets are against these attacks and if it is possible to obtain multiple modalities at the same time from one model. Another future direction to consider is if domain knowledge or a data driven approach can be used to make the search more efficient, thus reducing the number of required queries.

## Footnotes

1 Actiwatch, Cambridge Neurotechnology Ltd, England, model AW4
